# A single-nucleotide polymorphism in the 3′-UTR region of the adipocyte fatty acid binding protein 4 gene is associated with prognosis of triple-negative breast cancer

**DOI:** 10.18632/oncotarget.7920

**Published:** 2016-03-04

**Authors:** Wenmiao Wang, Peng Yuan, Dianke Yu, Feng Du, Anjie Zhu, Qing Li, Pin Zhang, Dongxin Lin, Binghe Xu

**Affiliations:** ^1^ Department of Medical Oncology, Cancer Institute and Hospital, Peking Union Medical College, Chinese Academy of Medical Sciences, Beijing, P.R. China; ^2^ Phase I Clinical Trial Center, Beijing Shijitan Hospital, Capital Medical University, The 9th Clinical College, Beijing, P.R. China; ^3^ Department of Etiology and Carcinogenesis, Cancer Institute and Hospital, Chinese Academy of Medical Science and Peking Union Medical College, Beijing, P.R. China

**Keywords:** triple-negative breast cancer, FABP4, genetic variant, 3′-untranslated regions, prognosis

## Abstract

Triple-negative breast cancer (TNBC) is a subtype of breast cancer with poor prognosis and high heterogeneity. The aim of this study was to screen patients for single-nucleotide polymorphisms (SNPs) associated with the prognosis of TNBC. Database-derived SNPs (NextBio, Ensembl, NCBI and MirSNP) located in the 3′-untranslated regions (3′-UTRs) of genes that are differentially expressed in breast cancer were selected. The possible associations between 111 SNPs and progression risk among 323 TNBC patients were investigated using a two-step case-control study with a discovery cohort (n=162) and a validation cohort (n=161). We identified the *rs1054135* SNP in the adipocyte fatty acid binding protein 4 (*FABP4*) gene as a predictor of TNBC recurrence. The *G* allele of *rs1054135* was associated with a reduced risk of disease progression as well as a prolonged disease-free survival time (DFS), with a hazard ratio (HR) for recurrence in the combined sample of 0.269 [95%CI: 0.098−0.735;*P*=0.001]. Notably, for individuals having the *rs1054135* SNP with the *AA/AG* genotype, the magnitude of increased tumour recurrence risk for overweight patients (BMI≥25kg/m^2^) was significantly elevated (HR2.53; 95%CI: 1.06–6.03). Immunohistochemical staining of adipocytes adjacent to TNBC tissues showed that the expression level of *FABP4* was statistically significantly lower in patients with the *rs1054135*-*GG* genotype and those in the disease-free group (*P*=0.0004 and *P*=0.0091, respectively). These results suggested that the expression of a lipid metabolism-related gene and an important SNP in the 3′-UTR of *FABP4* are associated with TNBC prognosis, which may aid in the screening of high-risk patients with TNBC recurrence and the development of novel chemotherapeutic agents.

## INTRODUCTION

Triple-negative breast cancers (TNBCs) are a diverse and heterogeneous group of tumours that, by definition, lack estrogen and progesterone receptors and amplification of the *HER2* (human epidermal growth factor receptor-2) gene [1–2]. The majority of the tumours classified as TNBCs are highly malignant, characterized by their aggressive behavior, young age of onset, and early relapse [3–4]. Transcriptional profiling studies suggest that there is further heterogeneity within triple-negative breast cancers, and these tumours can be categorized into six or more groups using genomic analysis [5–6]. However, the high expense of these detection methods and instability of their prognostic efficacy makes this profiling applicable only within laboratories. Therefore, more effective and sensitive prognostic markers are urgently needed to further subdivide TNBCs and to guide clinical practice more accurately.

A significant fraction of cancer patients have occult disseminated tumours at the time of primary diagnosis, which usually progress to clinically relevant lesions [7]. Since the majority of cancer mortality is associated with metastatic disease, biomarkers with the ability to predict metastatic risk in tumours would be of great value. Recent advances have led to the recognition that microRNAs (miRNAs) can act as key genetic regulators of a wide variety of biological processes, including tumour growth, proliferation, and survival [8–9]. Indeed, a number of miRNAs have been identified as potent oncogenes and tumour suppressors, playing crucial roles in the metastatic process of breast cancer [10–11]. More importantly, a series of studies has revealed strong correlations between altered miRNA expression and distant disease-free survival (DDFS), as well as overall survival (OS) of TNBC, suggesting their prognostic value for TNBC [12–13].

miRNAs are small, noncoding RNAs that regulate gene expression by degrading and/or suppressing the translation of target messenger RNAs (mRNA) by base pairing with sequences within the 3′-untranslated region (UTR) of mRNA [14]. On the one hand, miRNAs regulate gene expression in a post-transcriptional manner [15]. On the other hand, a reciprocal feedback loop between miRNAs and their target genes is often observed. Emerging evidence reveals that miRNA expression maybe regulated by single-nucleotide polymorphisms (SNPs) in the 3′-UTRs of their target genes [16–17]. In other words, SNPs located in the 3′-UTRs of target genes may influence not only the expression of the targeted genes but that of miRNAs as well. Therefore, these findings raise the possibility that some SNPs located in the complementary miRNA-binding sites of the 3′-UTRs of target genes may influence the biological properties of tumour cells through their impact on the expression of both targeted genes and miRNAs. Together, these may eventually determine individual susceptibility to tumour metastasis.

Although increasing evidence suggests that polymorphisms in such areas could act as strong predictors of cancer risk and prognosis, including that of breast cancer [18–20], none of the previously identified miRNA-altering polymorphisms have been specifically associated with the outcome of triple-negative breast cancer. Thus, in this case-control study, we identified the main SNPs located in the 3′-UTRs of differentially expressed genes in breast cancer in an attempt to discover the genetic variants in the 3′-UTR that potentially influence interactions with miRNAs and are associated with TNBC recurrence in a Chinese Han population.

## RESULTS

### Subject characteristics

The detection rates in 12 samples were less than 90% and were excluded from the final analysis of the discovery cohort. The overall median follow-up times of the discovery and validation cohorts were 89.8 and 47.3 months, respectively. Table 1 describes the characteristics of the study population. Surprisingly, the distributions of some clinical characteristics differed between the two cohorts. However, there was a consistently higher incidence of lymph node metastasis in the relapse group.

**Table 1:** Pretreatment characteristics of the discovery and validation cohorts

	Discovery cohort			Validation cohort		
	Disease –free n=113(%)	Relapse n=36(%)	*P^a^*	Disease –free n=114(%)	Relapse n=48(%)	*P^a^*
Age(y)			**0.012**			0.403
≦40	17(15.0)	12(33.3)		23(20.2)	7(14.6)	
>40	96(85.0)	24(66.7)		91(79.8)	41(85.4)	
BMI(Body Mass Index)			0.995			0.456
	25.02+-0.32	25.01+-0.62		24.04+-0.32	24.65+-0.75	
Breast cancer/Ovarian cancer history			1.000			0.750
yes	6(5.3)	2(5.6)		8(7.0)	4(8.3)	
no	107(94.7)	34(94.4)		106(93.0)	44(91.7)	
Menopausal status at diagnosis			**0.027**			0.395
premenopausal	72(63.7)	30(83.3)		63(55.3)	30(62.5)	
postmenopausal	41(36.3)	6(16.7)		51(44.7)	18(37.5)	
Operation method			0.887			0.421
modified radical mastectomy	93(82.3)	30(83.3)		86(75.4)	39(81.3)	
breast conserving surgery	20(17.7)	6(16.7)		28(24.6)	9(18.7)	
Histological type			0.404			0.438
infiltrative nonspecific cancer	88(85.4)	33(91.7)		107(93.9)	47(97.9)	
others	15(14.6)	3(8.3)		7(6.1)	1(2.1)	
Histological grade			0.299			0.342
I-II	46(49.5)	12(38.7)		46(47.4)	15(38.5)	
III	47(50.5)	19(61.3)		51(52.6)	24(61.5)	
unknown	19	5		17	9	
Lymphatic vessel invasion			0.190			0.082
yes	12(10.6)	1(2.8)		5(4.4)	6(12.8)	
no	101(89.4)	35(97.2)		109(95.6)	41(87.2)	
Unknown	0	0		0	1	
Tumour size			0.894			**0.036**
≦2cm	43(38.4)	13(37.1)		54(47.8)	14(29.8)	
>2cm	69(61.6)	22(62.9)		59(52.2)	33(70.2)	
Unknown	1	1		1	1	
Lymph-node involvement			**0.043**			**0.039**
no	74(66.1)	17(47.2)		76(67.3)	24(50.0)	
yes	38(33.9)	19(52.8)		37(32.7)	24(50.0)	
Unknown	1	0		1	0	
Taxane/anthracycline-based chemotherapy			0.303			0.454
no	12(10.7)	1(2.9)		5(4.5)	4(8.3)	
yes	100(89.3)	34(97.1)		107(95.5)	44(91.7)	
unknown	1	1		2	0	
Radiotherapy			0.097			0.671
no	68(60.2)	16(44.4)		42(36.8)	16(33.3)	
yes	45(39.8)	20(55.6)		72(63.2)	32(66.7)	

Two-sided χ^2^ test

### Genotyping and association analysis of SNPs and TNBC prognosis

To identify SNPs with potential prognostic value, 149 samples in the discovery cohort were tested initially. Sixteen SNPs were not in Hardy–Weinberg equilibrium (data not shown). Results from association analyses for 111 SNPs and the risk of disease progression are presented in Table 2. Fourteen SNPs were associated with the risk of TNBC recurrence and metastasis (*P*<0.05).

**Table 2:** Association between SNPs in differentially expressed genes and the risk of disease progression

Gene	SNP	Alleles (major/minor)	MAF		HR^a^ (95% CI)	*P*	Genetic model
			Disease-free	Relapse			
*CCND1*	*rs678653*	*G/C*	0.1	0.07	18.09 (0.66-495.58)	0.1	REC
*ESR1*	*rs3798577*	*T/C*	0.40	0.46	1.35 (0.50-3.60)	0.55	DOM
*ADAMTS1*	*rs2738*	*C/A*	0.17	0.08	0.46 (0.17-1.29)	0.12	ADD
*ADAMTS1*	*rs9636786*	*T/C*	0.17	0.21	1.21 (0.48-3.06)	0.69	DOM
*ADH1B*	*rs1042026*	*G/A*	0.27	0.29	0.44 (0.07-2.67)	0.35	REC
*ADH1B*	*rs17033*	*A/G*	0.14	0.11	0.77 (0.27-2.20)	0.63	DOM
*ATM*	*rs227092*	*G/T*	0.44	0.41	1.58 (0.44-5.68)	0.49	REC
*BACH1*	*rs15092*	*A/G*	0.08	0.06	0.00 (0.00-NA)	0.20	REC
*BRCA1*	*rs12516*	*C/T*	0.35	0.36	1.46 (0.42-5.05)	0.56	REC
*BRIP1*	*rs7213430*	*A/G*	0.31	0.39	2.14 (1.09-4.22)	0.026	ADD
*C8orf4*	*rs10199*	*A/G*	0.42	0.47	1.75 (0.91-3.34)	0.086	ADD
*CASP8*	*rs1045494*	*T/C*	0.18	0.24	1.44 (0.59-3.53)	0.42	DOM
*CCDC170*	*rs3734806*	*G/A*	0.39	0.42	1.43 (0.35-5.87)	0.62	REC
*CCDC170*	*rs3757322*	*T/G*	0.40	0.42	0.76 (0.29-2.01)	0.58	DOM
*CCDC170*	*rs9383935*	*C/T*	0.38	0.43	1.52 (0.39-5.90)	0.55	REC
*CCDC170*	*rs6932260*	*T/C*	0.50	0.42	0.53 (0.27-1.02)	0.052	ADD
*CCDC170*	*rs9383589*	*A/G*	0.36	0.32	0.44 (0.17-1.11)	0.078	DOM
*CCND1*	*rs7177*	*A/C*	0.12	0.06	19.20 (0.71-520.31)	0.093	REC
*CDH1*	*rs13689*	*T/C*	0.17	0.15	1.24 (0.27-5.82)	0.78	REC
*CDS1*	*rs6827228*	*C/T*	0.11	0.06	0.39 (0.10-1.50)	0.14	ADD
*CLDN5*	*rs12628900*	*C/T*	0.09	0.14	1.94 (0.69-5.43)	0.21	REC
*COL10A1*	*rs1059277*	*G/A*	0.02	0.03	1.66 (0.23-11.82)	0.62	REC
*COL11A1*	*rs9659030*	*T/C*	0.20	0.21	1.89 (0.77-4.65)	0.16	DOM
*COL1A1*	*rs1061947*	*C/T*	0.04	0.03	1.21 (0.16-8.93)	0.85	REC
*COL1A1*	*rs1061237*	*T/C*	0.48	0.46	0.43 (0.12-1.49)	0.16	REC
*COL4A2*	*rs1049977*	*T/C*	0.19	0.12	0.41 (0.15-1.16)	0.08	DOM
*CSMD1*	*rs583087*	*C/T*	0.06	0.07	1.36 (0.41-4.58)	062	REC
*CYYR1*	*rs17002176*	*A/G*	0.04	0.07	1.79 (0.44-7.32)	0.43	REC
*CYYR1*	*rs17002187*	*G/A*	0.04	0.07	2.97 (0.78-11.36)	0.12	REC
*CYYR1*	*rs219643*	*C/T*	0.00	0.00	0.00 (0.00-NA)	0.59	REC
*CYYR1*	*rs2830239*	*A/G*	0.29	0.32	0.39 (0.04-3.79)	0.38	REC
*ERBB4*	*rs1595064*	*C/G*	0.43	0.44	0.69 (0.20-2.44)	0.56	REC
*ERBB4*	*rs1595065*	*T/C*	0.28	0.29	1.25 (0.50-3.12)	0.64	DOM
*ERBB4*	*rs10932374*	*G/A*	0.30	0.36	1.76 (0.50-6.22)	0.39	REC
*ERBB4*	*rs1836724*	*T/C*	0.25	0.24	0.61 (0.05-6.90)	0.68	REC
*ERBB4*	*rs12467225*	*C/T*	0.33	0.26	0.54 (0.23-1.28)	0.16	DOM
*ERBB4*	*rs1972820*	*T/C*	0.27	0.21	0.66 (0.31-1.44)	0.29	ADD
*ERBB4*	*rs11895168*	*C/A*	0.28	0.25	0.76 (0.31-1.85)	0.54	DOM
*ERBB4*	*rs1595066*	*G/A*	0.33	0.33	0.81 (0.34-1.95)	0.64	DOM
*ERBB4*	*rs3748960*	*T/C*	0.07	0.14	3.51(1.07-11.46)	0.039	DOM
*ERBB4*	*rs4672612*	*G/A*	0.24	0.24	0.00 (0.00-NA)	0.19	REC
*ERBB4*	*rs16845990*	*T/C*	0.33	0.27	0.63 (0.23-1.73)	0.37	DOM
*ESR1*	*rs3798758*	*G/T*	0.30	0.26	2.81 (0.37-21.24)	0.33	REC
*ETV6*	*rs1062298*	*G/T*	0.42	0.44	1.38 (0.46-4.12)	0.56	REC
*ETV6*	*rs1573613*	*T/C*	0.46	0.53	1.35 (0.45-4.08)	0.59	DOM
*ETV6*	*rs2156932*	*A/G*	0.03	0.07	2.30 (0.55-9.53)	0.25	REC
*ETV6*	*rs1573612*	*T/C*	0.46	0.50	1.73 (0.54-5.54)	0.36	REC
***FABP4***	***rs1054135***	***A/G***	**0.47**	**0.33**	**0.35 (0.15-0.80)**	**0.0084**	**ADD**
*FBN1*	*rs11070641*	*T/C*	0.13	0.15	1.16 (0.47-2.84)	0.74	ADD
*FBN1*	*rs12050562*	*C/T*	0.22	0.39	2.54 (1.26-5.13)	0.0077	ADD
*FGFR2*	*rs1047057*	*C/T*	0.43	0.37	0.45 (0.11-1.81)	0.24	REC
*GNAI1*	*rs17153599*	*C/T*	0.11	0.15	1.27 (0.52-3.09)	0.6	ADD
*KRAS*	*rs9266*	*C/T*	0.26	0.25	0.68 (0.32-1.49)	0.33	ADD
*KRAS*	*rs1137282*	*T/C*	0.11	0.07	0.42 (0.13-1.39)	0.13	ADD
*KRAS*	*rs12587*	*C/A*	0.28	0.24	0.00 (0.00-NA)	0.091	REC
*KRAS*	*rs13096*	*G/A*	0.23	0.25	0.38 (0.04-3.62)	0.35	REC
*KRAS*	*rs7973450*	*A/G*	0.08	0.07	0.65 (0.20-2.16)	0.47	ADD
*KRAS*	*rs712*	*G/T*	0.25	0.18	0.00 (0.00-NA)	0.014	REC
*KRAS*	*rs7960917*	*T/C*	0.12	0.08	0.55 (0.19-1.56)	0.24	ADD
*KCNMB3*	*rs3976507*	*G/A*	0.07	0.09	1.00 (0.28-3.59)	1.00	DOM
*KHDRBS3*	*rs3184618*	*A/G*	0	0.01	2.52 (0.05-121.99)	0.64	REC
*MSRB3*	*rs7711*	*A/G*	0.16	0.13	0.93 (0.35-2.48)	0.89	REC
*MSRB3*	*rs7316024*	*T/A*	0.49	0.47	1.41 (0.50-3.99)	0.51	DOM
*NTRK2*	*rs11140793*	*A/C*	0.05	0.06	0.74 (0.15-3.78)	0.72	REC
*NTRK2*	*rs3654*	*A/G*	0.18	0.14	0.00 (0.00-NA)	0.046	REC
*NTRK2*	*rs2013566*	*A/G*	0.17	0.13	0.00 (0.00-NA)	0.058	REC
*NTRK2*	*rs3739570*	*T/C*	0.50	0.49	0.49 (0.18-1.32)	0.16	DOM
*NTRK2*	*rs1047896*	*T/C*	0.06	0.08	0.00 (0.00-NA)	0.49	REC
*NTRK2*	*rs1624327*	*C/T*	0.11	0.17	23.08 (0.46-NA)	0.11	REC
*NTRK2*	*rs1221*	*G/A*	0.05	0.04	0.18 (0.02-1.71)	0.18	REC
*NTRK2*	*rs1627784*	*A/G*	0.33	0.38	1.78 (0.52-6.06)	0.36	REC
*NTRK2*	*rs7020204*	*C/T*	0.16	0.15	0.78 (0.35-1.76)	0.54	ADD
*NTRK2*	*rs3780634*	*A/G*	0.07	0.04	0.15 (0.02-1.35)	0.04	REC
*NTRK2*	*rs10780691*	*C/T*	0.3	0.2	0.00(0.00-NA)	0.04	REC
*NTRK2*	*rs7816*	*T/A*	0.09	0.21	3.67 (1.41-9.60)	0.0064	ADD
*PHB*	*rs1049620*	*A/G*	0.43	0.56	1.81 (0.85-3.83)	0.12	ADD
*PID1*	*rs3771286*	*C/T*	0.52	0.35	0.34 (0.16-0.76)	0.0051	ADD
*RHOU*	*rs1062060*	*C/T*	0.05	0.07	0.66 (0.15-2.96)	0.58	DOM
*RHOU*	*rs13349*	*A/G*	0.24	0.26	0.30 (0.03-3.00)	0.26	REC
*RHOU*	*rs11578216*	*T/A*	0.06	0.04	1.05 (0.23-4.66)	0.95	DOM
*RHOU*	*rs11580020*	*G/A*	0.07	0.04	0.00 (0.00-NA)	0.69	ADD
*RHOU*	*rs2058703*	*T/C*	0.01	0.03	2.30 (0.31-17.13)	0.43	DOM
*RND3*	*rs10185950*	*A/C*	0.07	0.03	0.23 (0.03-2.04)	0.13	DOM
*SASH1*	*rs8641*	*A/G*	0.33	0.31	1.84 (0.35-9.68)	0.48	REC
*SFRP1*	*rs3242*	*C/T*	0.05	0.04	1.39 (0.31-6.23)	0.67	REC
*SLC24A2*	*rs3739481*	*G/C*	0.49	0.54	0.66 (0.24-1.78)	0.41	DOM
*SLC24A2*	*rs4977544*	*C/T*	0.09	0.04	0.26 (0.06-1.10)	0.045	REC
*SLC24A2*	*rs4977545*	*G/T*	0.22	0.13	0.50 (0.20-1.22)	0.11	ADD
*SLC24A2*	*rs7864646*	*A/G*	0.07	0.04	0.66 (0.15-2.98)	0.58	REC
*SLC24A2*	*rs7872265*	*T/C*	0.46	0.36	0.56 (0.23-1.37)	0.20	DOM
*SLC24A2*	*rs7867513*	*C/T*	0.35	0.34	1.72 (0.43-6.90)	0.46	REC
*SLC24A2*	*rs7022987*	*C/T*	0.33	0.4	0.68 (0.26-1.75)	0.42	DOM
*SLC24A2*	*rs7854673*	*A/T*	0.07	0.00	0.00 (0.00-NA)	8e-04	REC
*SLC24A2*	*rs1556000*	*G/T*	0.08	0.05	0.67 (0.15-2.88)	0.58	REC
*SORCS1*	*rs12359404*	*C/T*	0.13	0.13	13.15 (0.43-406.54)	0.16	REC
*SORCS1*	*rs10491050*	*T/C*	0.22	0.25	1.36 (0.55-3.33)	0.51	DOM
*SORCS1*	*rs11192963*	*T/C*	0.26	0.33	2.06 (0.95-4.49)	0.065	ADD
*TAB2*	*rs2744434*	*G/A*	0.41	0.53	1.52 (0.78-2.95)	0.22	ADD
*TAB2*	*rs7896*	*C/G*	0.08	0.09	NA (0.00-NA)	0.011	REC
*TACSTD2*	*rs7333*	*G/A*	0.15	0.12	0.54(0.19-1.53)	0.23	ADD
*THSD4*	*rs12594531*	*C/A*	0.37	0.43	2.21 (0.80-6.11)	0.11	DOM
*THSD4*	*rs3087532*	*C/T*	0.18	0.15	0.00 (0.00-NA)	0.088	REC
*THSD4*	*rs7402189*	*A/G*	0.24	0.21	1.46 (0.59-3.60)	0.41	DOM
*THSD4*	*rs10468050*	*G/C*	0.13	0.11	0.84 (0.28-2.53)	0.76	DOM
*THSD4*	*rs1054260*	*C/T*	0.25	0.19	0.51 (0.23-1.10)	0.075	ADD
*THSD4*	*rs4776575*	*G/A*	0.20	0.20	1.81 (0.16-19.79)	0.63	REC
*TPM1*	*rs6738*	*A/G*	0.05	0.03	0.47 (0.09-2.50)	0.35	REC
*TPM1*	*rs7178040*	*G/T*	0.02	0.00	0.00 (0.00-NA)	0.13	REC
*ZNF365*	*rs11819488*	*A/G*	0.19	0.31	2.74 (1.28-5.86)	0.0075	ADD
*ZNF365*	*rs729739*	*G/A*	0.09	0.06	0.00 (0.00-NA)	0.41	REC
*ZNF365*	*rs729738*	*C/A*	0.08	0.07	1.18 (0.34-4.09)	0.79	REC

Hazard ratios (HRs) and 95% confidence intervals (CIs) for the association between SNPs and recurrence & metastasis risk was adjusted for age, tumour size (≤2cm and >2 cm), lymph-node involvement (no and yes), histological type, menopausal status (no and yes), vascular invasion (no and yes), breast or ovarian cancer history (no and yes), taxane/anthracycline-based chemotherapy (yes or no) and radiotherapy (no and yes)

Independent validation was conducted in the second cohort, which included 162 TNBCs with 114 disease-free cases and 48 relapsed cases. Three SNPs were significantly associated with TNBC recurrence (*P*<0.05), including *FABP4 rs1054135*, *KRAS* rs712 and *NTRK2* rs7816 (Table 3). The statistical significance was retained after multiple comparisons only for *FABP4rs1054135*. The *G* allele of rs1054135 was associated with a reduced risk of disease progression with an adjusted hazard ratio of 0.14(0.03-0.66) in a recessive model (Table 4).

**Table 3:** Association of SNPs rs1054135, rs712, rs7816, rs12050562, rs3748960, rs3654, rs11819488, rs10780691, rs7213430, rs3771286, rs4977544, rs7896, rs7854673, rs3780634, and the risk of disease progression

Gene	SNP	Alleles (major/minor)	MAF		HR^a^ (95% CI)	*P*	Genetic model	FDR
			Disease-free	Relapse				
*FABP4*	*rs1054135*	*A/G*	0.48	0.32	0.36 (0.19-0.69)	**0.0012**	ADD	**0.017**
*KRAS*	*rs712*	*G/T*	0.21	0.33	2.11 (1.12-3.95)	**0.019**	ADD	0.247
*NTRK2*	*rs7816*	*T/A*	0.18	0.14	0.00 (0.00-NA)	**0.031**	REC	0.372
*FBN1*	*rs12050562*	*C/T*	0.24	0.27	2.00 (0.92-4.36)	0.077	DOM	0.847
*ERBB4*	*rs3748960*	*T/C*	0.08	0.02	0.36 (0.08-1.61)	0.12	ADD	1.20
*NTRK2*	*rs3654*	*A/G*	0.17	0.22	1.55 (0.77-3.12)	0.22	ADD	1.98
*ZNF365*	*rs11819488*	*A/G*	0.19	0.21	2.56 (0.31-21.06)	0.39	REC	3.12
*NTRK2*	*rs10780691*	*C/T*	0.22	0.24	0.50 (0.09-2.90)	0.42	REC	2.94
*BRIP1*	*rs7213430*	*A/G*	0.28	0.26	0.83 (0.47-1.47)	0.52	ADD	3.12
*PID1*	*rs3771286*	*C/T*	0.5	0.45	0.85 (0.49-1.45)	0.54	ADD	2.70
*SLC24A2*	*rs4977544*	*C/T*	0.05	0.03	0.69 (0.17-2.82)	0.59	REC	2.26
*TAB2*	*rs7896*	*C/G*	0.06	0.05	0.64 (0.06-6.86)	0.71	REC	2.13
*SLC24A2*	*rs7854673*	*A/T*	0.06	0.06	1.25 (0.37-4.20)	0.72	REC	1.44
*NTRK2*	*rs3780634*	*A/G*	0.05	0.04	1.13 (0.30-4.31)	0.85	REC	0.85

Hazard ratios (HRs) and 95% confidence intervals (CIs) for the association between SNPs and recurrence & metastasis risk was adjusted for age, tumour size (≤2cm and >2 cm), lymph-node involvement (no and yes), histological type, menopausal status (no and yes), vascular invasion (no and yes), breast or ovarian cancer history (no and yes), taxane/anthracycline-based chemotherapy (yes or no) and radiotherapy (no and yes)

**Table 4:** Association between the *rs1054135* genotype and the risk of TNBC relapse (validation cohort)

SNP	genotype	Disease-free (%)	Relapse (%)	HR^a^ (95% CI)	*P* value
*rs1054135*	*AA+AG*	88(80.0)	42 (95.5)	1.00(Reference)	
	*GG*	22 (20.0)	2(4.5)	0.14(0.03-0.66)	0.0026

Hazard ratios (HRs) and 95% confidence intervals (CIs) for the association between SNPs and recurrence & metastasis risk was adjusted for age, tumour size (≤2cm and >2 cm), lymph-node involvement (no and yes), histological type, menopausal status (no and yes), vascular invasion (no and yes), breast or ovarian cancer history (no and yes), taxane/anthracycline-based chemotherapy (yes or no) and radiotherapy (no and yes).

The data also showed a covariate effect of BMI and *FABP4* on TNBC reccurence. For patients with the *AA/AG* genotype, the magnitude of increased tumour recurrence risk for overweight patients (BMI≥25kg/m^2^) was significantly elevated (HR, 2.53; 95%CI, 1.06–6.03).

**Figure 1:**
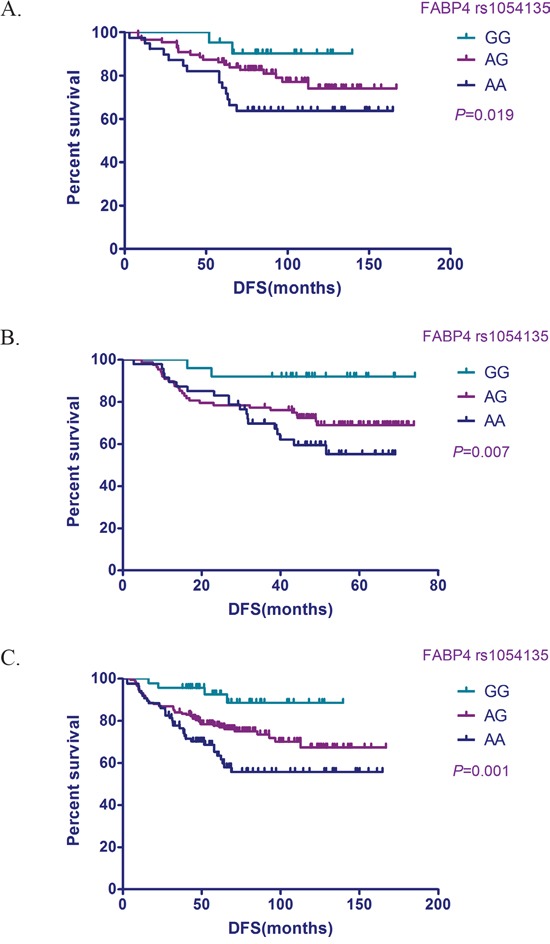
Relationship between the *FABP4* SNP *rs1054135* and DFS in TNBC patients Kaplan–Meier survival probability plots stratified by *FABP4rs1054135* genotype. **A.** Discovery cohort. **B.** Validation cohort. **C.** Combined sample.

Positive results were obtained for the associations between the *rs1054135* genotype and DFS using the Kaplan–Meier method (Figure 1). The discovery and validation sets were subsequently combined for analysis, and showed that individuals with the *rs1054135*-*GG* genotype were associated with a prolonged DFS, with a HR of 0.269 (95%CI = 0.098−0.735; *P*= 0.001).

### Correlations between *FABP4* expression, the *rs1054135* genotype and TNBC prognosis

In order to test the hypothesis that the *rs1054135* genotype facilitates tumour metastasis by regulating *FABP4* expression, its protein expression was analyzed by immunohistochemistry in 52 TNBC tissues (disease-free group, n=34; relapsed group, n=18) with the associated genotype data.

**Figure 2:**
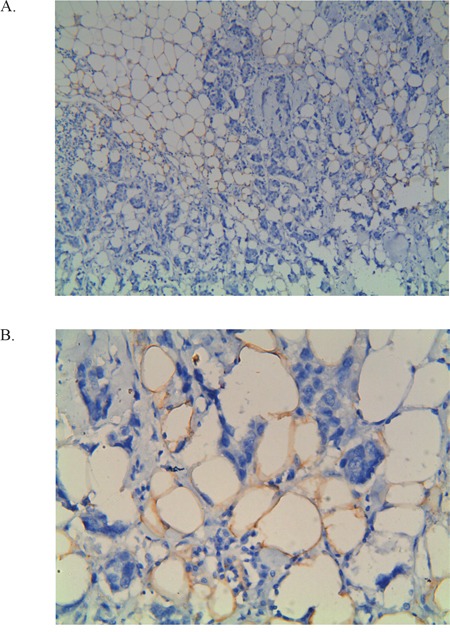
Example of immunohistochemical staining with *FABP4* **A.** 100x magnification and the same area at 400x magnification **B.** The IOD counts were performed by the computer using 400x magnification of these images.

**Figure 3:**
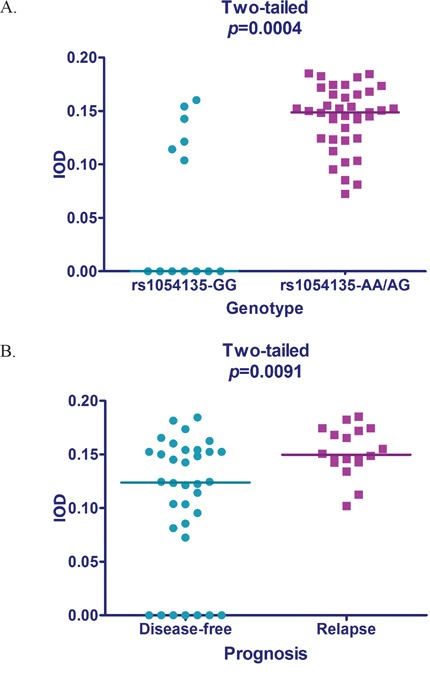
Scatterplot of *FABP4* expression **A.** Intergroup difference of *FABP4* expression in patients with different prognosis tested by the Mann-Whitney U test. **B.** Intergroup difference of *FABP4* expression associated with different *rs1054135* genotypes tested by the Mann-Whitney U test.

Remarkably, adipocytes adjacent to breast tissue exhibited higher *FABP4* protein levels, when compared with those located more distantly from breast tissues (Figure 2). Quantitative determination with IOD also showed that *FABP4* protein levels were significantly higher in adipocytes with the *rs1054135*-*AA/AG* genotype (*P*<0.01). The median IOD of the *rs1054135*-*AA/AG* group was 0.149, while more than half of patients with the *rs1054135*-*GG* genotype did not express *FABP4* (IOD=0). A significant difference in *FABP4* protein levels was also observed between the disease-free and relapse groups (with a median IOD of 0.1240 and 0.1498, respectively), which is consistent with our hypothesis (Figure 3).

## DISCUSSION

The strong invasiveness of TNBCs is manifested in an early onset of recurrence and metastasis, particularly in the first three years [21]. In our study, 60% of patients in the relapse group suffered disease progression within three years, and the five-year DFS of the total sample was 75.7%. These results are similar to those previously reported for TNBC studies; however, they are significantly worse than those reported for other subtypes [22]. Therefore, the identification of TNBC subgroups relevant to clinical prognosis will aid in the design and administration of individualized treatment plans.

By a two-stage analysis of discovery and validation samples, we identified a novel variant in FABP4 associated with both recurrence risk and DFS of TNBC. In our study, we found that the A allele of rs1054135 could upregulate FABP4 expression in TNBC patients, which was in line with the findings of a previous study that in children with obstructive sleep apnea, the rs1054135 AA genotype was associated with high serum FABP4 levels [23], suggesting a functional relevance of this site.

According to bioinformatics predictions, rs1054135 was a miR-3685 complementary SNP site and a G>A transition at rs1054135 may lead to an increased binding force with miR-3685. Scientists have demonstrated that, in some mRNAs with AU-rich elements (AREs), miRNAs could mediate a direct association of micro-ribonucleoproteins (microRNPs) with the AREs and eventually upregulate translation in some cases [24]. Likewise, variation in rs1054135 may affect the expression of FABP4 through similar molecular mechanisms, while further investigations are needed to validate this speculation.

Adipocyte fatty acid binding protein4 (*FABP4*) is predominantly expressed in the cytosol of mature adipocytes and reversibly binds long-chain fatty acids. Previous reports have characterized its role in lipid metabolism and transport [25]. *In vitro* studies showed that cocultivation of several cancer cell lines (ovarian, breast, and colon) with adipocytes induced *FABP4* mRNA expression. Controversially, when adding a *FABP4* inhibitor to a coculture of ovarian cancer cells and adipocytes, lipid accumulation in the cancer cells and adipocyte-mediated invasion were drastically reduced [26]. Similarly, in our study, stronger immunohistochemical (IHC) staining of *FABP4* was observed in adipocytes adjacent to breast tissue, implying that *FABP4* may function as a mediator of lipid trafficking, and the expression level of *FABP4* maybe an indicator of the regional metabolic level. Moreover, FABP4 was found to be induced by VEGFA and/or the NOTCH pathway in endothelial cells, and inhibition of FABP4 blocks most of the VEGFA effects, suggesting its role in tumour angiogenesis [27]. Nieman KM, et al. reported an up regulation of *FABP4* expression in metastatic human ovarian cancer samples compared with primary ovarian tumours; the increased *FABP4* levels were shown to fuel rapid tumour growth and support metastasis [28]. Furthermore, previous studies have identified FABP4 as a prognostic marker in breast cancer. Hancke K et al. found that higher serum *FABP4* levels were associated with obese breast cancer, as well as greater tumour size and lymph node involvement [29]. A most recent report also showed that *FABP4* positivity was associated with significantly shorter DFS and OS in TNBC [30]. However, unlike in our study, tumour tissues instead of stroma were used as the IHC target, and the positive rate of *FABP4* was relatively low (2/50), making it a less statistically powerful prognostic biomarker.

Our study found a closed-loop chain between rs1054135, FABP4 expression and TNBC prognosis. Given that FABP4 is a significant medium of fuel supply for tumour growth, and probably involved in tumour angiogenesis, the rs1054135 SNP located in the 3′-UTR of FABP4 may influence patient susceptibility to TNBC recurrence through posttranscriptional regulation of FABP4 expression.

Indeed, the roles of lipid metabolism-related genes and pathways in tumour development have been studied extensively, especially in breast cancer. Several studies indicated that adipose tissue itself is an endocrine organ that could influence tumour growth or differentiation via adipose tissue-derived hormones [31] called adipocytokines, e.g., leptin, resistin, or adiponectin (ApN), most of which showed strong correlations with BMI [32–33]. However, the association between obesity and survival after breast cancer remained controversial for decades until last year; a positive association was demonstrated in meta-analyses of published data [34–35]. In the present study, a positive association between obesity and high recurrence risk was observed for the *rs1054135*-*AA/AG* subgroup. This provides additional evidence that body fat content and *FABP4* (as key substrates and enzymes of fat metabolism) functioned synergistically when fueling rapid tumour growth and metastasis. Therefore, it is safe to assume that the previous controversy over the association between BMI and breast cancer prognosis maybe related to the distribution difference of the *FABP4* genotype among different populations.

Recently, the Women's Intervention Nutrition Study (WINS) revealed that a low-fat diet after diagnosis of early breast cancer can reduce the death rate by 56%for women with both ER- and PR-negative breast cancer [36]. In addition, another large retrospective study reported that statin use was associated with a significant reduction in deaths from breast cancer (aHR = 0.60) [37] and, most importantly, statins were found to suppress the expression of FABP4 by previous basic research [38]. Thus, these findings shed light on the possibility that for obese patients with the *rs1054135*-*AA/AG* genotype, a low fat diet and statins could be selectively administered.

To our knowledge, our study is the first to examine the association of TNBC prognosis and SNPs located in the complementary miRNA binding sites of the 3′-UTRs of target genes. Since germline SNP variations are more stable than somatic SNP mutations, the germline SNP prognostic signature may provide more reliable information on individual susceptibility to tumour metastasis and be less likely to be affected by intratumour heterogeneity. Additionally, our findings are important because TNBC patients have fewer immediate therapeutic options, and these patients tend to have more aggressive disease. This study not only demonstrated the significant role of lipid metabolism in the process of TNBC recurrence, but discovered a novel SNP located in the 3′-UTR of *FABP4* that acted in concert with BMI and showed a strong association with DFS. Thus, for patients with the *rs1054135*-*AA/AG* genotype, low-fat diet intervention and body weight management is strongly recommended. More importantly, these results suggest that cutting off the ‘fuel supply’ may be a promising method for tumours such as TNBC that lack therapeutic targets.

However, despite the aforementioned strengths, we also acknowledge the limitations of this study. TNBC selection was based on immunohistochemistry instead of genomic analysis. Thus, a small proportion of other subtypes may have been involved. However, this confounding factor could hardly restrict the application, given that IHC diagnosis is still the gold standard in clinical practice. The second limitation is the sample size, which may have resulted in our study having limited statistical power. However, a two-step screening and validation process as well as the multiple-testing procedure were used to reduce the false-positive rate, and the validity of our results can be confirmed in future studies. Another limitation is the unequal follow-up time between the discovery and validation cohorts. Given that the primary endpoint of our study was DFS, and that TNBC patients have the highest percentage of early relapse, the relatively longer follow-up time in the discovery cohort was considered mainly due to the long-term observation of patients in the disease-free group. Therefore, it is well-founded to regard the difference in follow-up time between two cohorts as acceptable.

In conclusion, our study identified a lipid metabolism-related gene and an important SNP in the 3′-UTR of *FABP4* associated with TNBC prognosis, which may aid in the screening of high-risk patients with TNBC recurrence and the development of novel chemotherapeutic agents.

## MATERIALS AND METHODS

### Ethics statement

This investigation was conducted in accordance with the ethical standards of the Declaration of Helsinki and following the national and international guidelines and has been approved by the Institutional Review Board of the Chinese Academy of Medical Sciences Cancer Hospital.

### Study subjects

Tumour tissues and blood samples have been collected from primary breast cancer patients treated in our hospital since 1998, and there are a total of 13,240 blood samples. In the present study, we reviewed all of the pathologically confirmed TNBC cases from this sample library (n=430). Patients with a previous history of cancer (n=4) and insufficient blood samples (n=23) were excluded. Additionally, disease-free survivors with a follow-up time of less than three years were also excluded (n=80). Thus, a total of 323 TNBC patients were included in the final analysis. We artificially designated the date of Jan 1, 2008 a cut-off; patients diagnosed with TNBCs before Jan 1, 2008 were grouped into the discovery cohort (n=161) and those diagnosed after that date were grouped into the validation cohort (n=162). Patients were followed until April 1, 2014 to collect data on clinicopathological features, treatments, and vital status, such as recurrence and death. The DFS time was defined as the time from the date of surgery until the date of the first locoregional recurrence, first distant metastasis, or death from any cause. Patients known to be alive with no evidence of disease progression were censored at the last follow-up date or on April 1, 2014 (whichever came first).

ER (estrogen receptor) and PR (progesterone receptor) status was evaluated based on the IHC results of formalin-fixed, paraffin-embedded breast cancer tissue samples obtained from the patients. A positive ER and PR status was defined by nuclear staining of more than 1% according to guidelines issued by the American Society of Clinical Oncology (ASCO) and the College of American Pathologists (CAP) in 2010. Tumours negative for ER, PR, and HER2 were defined as TNBCs. However, there is growing evidence that low-HR-staining tumours (1%-10%) are clinicopathologically more similar to HR-negative than to HR-positive tumours [39]. Therefore, tumours with low and/or focal PR staining were included in our study. The IHC was performed with anti-ER and anti-PR antibodies. To determine the HER2 status, IHC or gene amplification was performed by fluorescence in situ hybridization (FISH).

### SNP selection and genotyping

SNP selection was a process taking full advantage of online databases and can be described as follows. First, the top 100 genes known to be differentially expressed in breast cancer were downloaded from the NextBio database (www.nextbio.com), searching using the keyword “breast cancer”. Second, these candidate genes were entered into the Ensembl (http://www.ensembl.org/index.html) and NCBI (http://hapmap.ncbi.nlm.nih.gov/) databases to select SNPs located in the 3′-UTRs of these differentially expressed genes with a minor allele frequency (MAF)≥ 5% in the ethnic Han Chinese people (n=204). Lastly, the MirSNP (http://cmbi.bjmu.edu.cn/mirsnp) publicly available online database was used for the final screening [40]. MirSNP contains a collection of human SNPs in predicted miRNA-mRNA binding sites, and 414,510 SNPs were identified to affect miRNA-mRNA binding through a miRNA target prediction algorithm, miRanda. In other words, only those SNPs with potential effects on miRNA-mRNA binding were included (n=140). Primers and probes were designed using MassARRAY Typer 4.0 software. In addition, 29 SNPs were excluded due to interference with primer binding. Therefore, 111 SNPs were included in the final genotyping.

For purposes of economy and efficiency, genotyping of the combined samples (n=323) was conducted using the MassARRAY MALDI-TOF System (Sequenom Inc., San Diego, CA, USA) at once by the method described in the Sequenom Genotyping Protocol, while association studies for the individual cohorts were analyzed separately. Duplicate samples and negative controls (without DNA) were included for quality assurance of genotyping. Concordance for duplicate samples was 100% for all assays. The analysts who carried out the genotyping were blinded to the group information on each sample.

### Immunohistochemistry

IHC staining of *FABP4* was performed on formalin-fixed, paraffin-embedded tissue sections. As *FABP4* is primarily expressed in the cytosol of mature adipocytes, we chose adipocytes adjacent to tumour tissues as targets. Briefly, 4-μm-thick sections were cut with a microtome, transferred onto adhesive slides, and then dried at 62°C for 15 min. All slides were incubated with primary antibody (*FABP4*, 1:100, ab92501, Abcam, Cambridge, UK). After applying primary antibodies, the tissues were incubated in blocking solution for 1 h at 37°C. Subsequently, immunodetection was performed using a commercial streptavidin-biotin kit according to the manufacturer's instructions, which involved incubation with biotinylated anti-mouse or anti-rabbit immunoglobulin, followed by peroxidase-labelled streptavidin and 3, 3′-diaminobenzidine chromogenic substrate. The primary antibody incubation step was omitted from the negative control. Finally, the slides were counterstained with Harris haematoxylin.

### Integrated optical density (IOD)

Using the Moticcam 2306^®^ 4 tablet (MOTIC Company Ltd., China), cellular membranes of adipocytes adjacent to breast tissues were selected at random from the digitized IHC images and their contours were precisely delineated with an Intuos pen using the selecting tool available within the ImageJ software (Image-Pro Plus 6.0). The contours of cellular membranes were transformed into vectorial masks and saved as TIFF format files. The latter were subjected to an ImageJ algorithm, which computed individual membrane area and associated IODs of the *FABP4* staining. The technicians were blinded to group information and SNP data for each sample.

### Statistical analyses

The differences in patients' characteristics for study inclusion were assessed by Pearson's χ^2^ tests, and all *P* values represent two-sided statistical tests. The continuous variable BMI with a normal distribution was expressed as a mean, and the intergroup difference was tested using the unpaired t-test. As for the IOD, a Shapiro–Wilk analysis was employed to validate the distribution characteristic, and a t-test or Mann-Whitney U test was selected for intergroup difference assessment, as appropriate. A P value of less than 0.05 was considered to indicate significance. The Kaplan-Meier and Cox methods were used to estimate the survival function stratified by genotype of the studied genes. Differences across survival curves were examined using a log-rank test.

For individual SNP analysis, we tested three genetic models (additive, dominant, and recessive) to evaluate the significance of SNPs, and the best-fitting model for each SNP was selected by the smallest *P* value. Hazard ratios (HRs) and 95% confidence intervals (CIs) for the association of SNPs and risk of recurrence and metastasis were adjusted for age, tumour size (≤2cm and >2 cm), lymph-node involvement (no and yes), histological type, menopausal status (no and yes), vascular invasion (no and yes), breast or ovarian cancer history (no and yes), taxane/anthracycline-based chemotherapy (yes or no), and radiotherapy (no and yes). Given the number of SNPs investigated, the Benjamini-Hochberg false discovery rate (FDR) method was used to assess statistical significance after correction for multiple comparisons. We considered an FDR of <0.05 to be noteworthy [41]. Tests for Hardy–Weinberg equilibrium were conducted. All statistical procedures were conducted using SPSS software (version 19.0) and GraphPad Prism5.
